# Outcomes from colonoscopy following referral from New Zealand general practice: a retrospective analysis

**DOI:** 10.1186/s12876-021-02042-7

**Published:** 2021-12-15

**Authors:** Ross Lawrenson, Sheena Moosa, Judy Warren, Ralph van Dalen, Lynne Chepulis, Tania Blackmore, Chunhuan Lao, Christopher Mayo, Jacquie Kidd, Melissa Firth, Tim Stokes, Mark Elwood, David Weller, Jon Emery

**Affiliations:** 1 Medical Research Centre, University of Waikato, Hamilton, New Zealand; 2 Waikato District Health Board, Hamilton, New Zealand; 3 Auckland University of Technology, Auckland, New Zealand; 4 Department of General Practice and Rural Health, University of Otago, Dunedin, New Zealand; 5 School of Population Health, University of Auckland, Auckland, New Zealand; 6 Centre for Population Health Sciences, The University of Edinburgh, Edinburgh, Scotland, UK; 7 Medicine, Dentistry and Health Sciences, The University of Melbourne, Melbourne, Australia

**Keywords:** Colorectal cancer, Colonoscopy rates, General practice, High suspicion of cancer

## Abstract

**Background:**

New Zealand has high rates of colorectal cancer (CRC) but poor outcomes. Most patients with CRC are diagnosed following referral from general practice, where a general practitioner (GP) assesses symptoms according to national guidelines. All referred patients are then re-prioritised by the hospital system. The first objective of this study was to identify what proportion of patients referred by general practice to surgical/gastroenterology at Waikato District Health Board (DHB) had a colonoscopy. The second objective was to determine what proportion of these referrals have an underlying CRC and the factors associated with the likelihood of this diagnosis.

**Methods:**

This study is a retrospective analysis of e-referral data for patients aged 30–70+ who were referred from 75 general practices to general surgery, gastroenterology or direct to colonoscopy at Waikato DHB, 01 January 2015–31 December 2017. Primary and secondary outcome measures included the proportion and characteristics of patients who were having colonoscopy, and of those, who were diagnosed with CRC. Data were analysed using chi square and logistic regression.

**Results:**

6718/20648 (32.5%) patients had a colonoscopy and 372 (5.5%) of these were diagnosed with CRC. The probability of having CRC following a colonoscopy increased with age (*p* value < 0.001). Females (*p* value < 0.001), non-Māori (*p* value < 0.001), and patients with a high suspicion of cancer (HSCan) label originating from their GP were more likely to have a colonoscopy, while the odds ratio of Māori having a colonoscopy was 0.66 (95% CI 0.60–0.73). The odds ratio of a CRC diagnosis following colonoscopy was 1.67 (95% CI 1.35–2.07) for men compared to women, and 2.34 (95% CI 1.70–3.22) for those with a GP HSCan label. Of the 585 patients referred with a GP HSCan, 423 (72.3%) were reprioritised by the hospital and 55 patients had their diagnosis unnecessarily delayed.

**Conclusions:**

If a GP refers a patient with an HSCan, and the patient receives a colonoscopy, then the likelihood of having CRC is almost 15.0%. This would suggest that these patients should be routinely prioritised without further triage by the hospital. Further research is needed to understand why Māori are less likely to receive a colonoscopy following referral from general practice.

## Background

Each year, approximately 1200 people in New Zealand (NZ) die of colorectal cancer (CRC) [1]. CRC occurs less frequently in Māori—the indigenous peoples of NZ prior to colonisation—compared to non-Māori [2]. Age, male gender, a family history and a raised body mass index (BMI) are recognised risk factors for CRC [3]. A personal history of adenomatous polyps or inflammatory bowel disease also increases risk [4]. Most patients with CRC are diagnosed following referral to a public hospital from a general practitioner (GP) [5, 6], who are asked to follow NZ Ministry of Health (MOH) guidelines for specialist referral of patients with signs and symptoms of bowel cancer. These guidelines outline symptoms that can include: rectal bleeding, blood mixed with stool; change in bowel habit (for at least 6 weeks); abdominal pain or bloating; weight loss and anaemia [7]. Patients with these symptoms usually present to their GP who will then arrange investigations and referral to specialist services [8]. More recently, direct access referral has been made available for GPs to refer patients for colonoscopy—again within strict guidelines outlining symptoms such as unexplained rectal bleeding with iron deficiency anaemia, changed bowel habit, family history or known/suspected CRC [9]. In certain defined circumstances such as persistent rectal bleeding or a change in bowel habit GPs can indicate a High Suspicion of Cancer (HSCan), and under current NZ guidelines these patients should be seen urgently within 2 weeks. However, under the NZ HSCan guidelines these referrals would still be triaged by hospital specialist services who make the final decision as to whether the referral is deemed high suspicion or can be considered for semi-urgent or routine follow up.

Referral from general practice for a diagnosis of suspected bowel cancer and colonoscopy is a relatively rare event. With 3100 new cases of CRC each year [1], on average the 3700 NZ GPs will see less than one new case of CRC per year—consistent with United Kingdom (UK) figures [10]. A Dutch study of 140,000 patients suggested only 2.0% of patients were referred for investigation of suspected CRC in a 5 year period [11]. What has not been widely reported is what proportion of patients referred for colonoscopy have an underlying cancer. Prior to the recent inauguration of a nationwide bowel cancer screening programme for 60–74 year olds, a screening pilot [12] that involved 50–74 year olds, carried out by Waitematā District Health Board (DHB) reported 212 new cancers found after 4500 colonoscopies following a positive Faecal Immunochemical test (FIT) result, or 4.7%. In a small study of 144 symptomatic patients with constipation from South Africa, it was found that 9/144 (6.25%) had an underlying CRC [13]. In Koning’s [11] general practice study only 2.0% (57/2785) of the patients who had a colonoscopy were diagnosed with CRC.

The Waikato DHB has a population of 430,000 and is located in the North Island of NZ. Twenty three percent of the Waikato population identify as Māori. The majority of cancer patients are managed in the public hospital system which is free to all NZ residents, but a small proportion of CRC patients may be seen and managed in the private health system. This study only relates to patients referred to the public health system. While generally all referrals are reviewed to see whether they will be offered as First Specialist Assessment (FSA), since 2016, for patients who have clear cut symptoms and are in the appropriate age range (> 50 years), GPs in the region have been able to make a direct referral for colonoscopy. However, these patients also require the approval of a specialist before a colonoscopy is arranged. Waikato DHB covers 75 general practices and it has been noted that the referral rates from practices vary greatly. It has been postulated that there is a correlation between referral rates and the risk of underlying pathology e.g., high referrers may have a lower positivity rate. It has been noted in the UK that using routine data on detection and conversion rates of different GPs should be interpreted with caution and is altered by the case mix of patients presenting [14]. The aim of our study is to identify the characteristics of patients aged 30–70+ referred to general surgical and gastroenterology outpatients (including direct referral) receiving a colonoscopy. We also wanted to establish risk of underlying CRC in those who had a colonoscopy and the factors associated with the likelihood of this diagnosis.

## Methods

The population investigated were patients aged 30–70+ referred from 75 general practices to general surgery, gastroenterology or direct to colonoscopy at Waikato DHB from 01 January 2015 to 31 December 2017. Data were sourced from Waikato DHBs electronic referral system, where referrals are generated from general practice following assessment of patient symptoms according to MOH referral guidelines. The extracted dataset included patient age, gender, ethnicity, date of referral, whether the patient had colonoscopy, whether it was direct access colonoscopy, whether the general practice was a high referrer (defined as a referral rate above the median referral rate), GP label of HSCan, and the hospital triage label of HSCan (i.e., specialist assessment of risk based on information supplied by the GP on the e-referral form. This risk assessment of all patients is undertaken by the hospital irrespective of the GP recommendation). This dataset was then linked to the National Cancer Register through the National Health Index (NHI) number (a unique identifier for all who use health and disability services in NZ) to identify any cancer diagnosis for the referred patients from 01 January 2015 to 31 December 2018. Ethical approval was obtained from the Health and Disability Ethic Committee of New Zealand (Approval Number: 17/NTB/156).

The characteristics of patients who received a colonoscopy were analysed and compared to the characteristics of patients who did not receive a colonoscopy. The difference was examined with a Chi-square test. Logistic regression was used to estimate the adjusted odds ratio (OR) and the 95% confidence interval (CI) of the odds ratio for these factors in the likelihood of colonoscopy.

We then analysed which patients were diagnosed with CRC following a colonoscopy. The characteristics of patients who had CRC were compared to patients who did not have CRC. Logistic regression was used to estimate the adjusted odds ratio for these factors in the likelihood of a CRC diagnosis. Cancer extent was described by colon cancer and rectal cancer. All data analyses were performed in IBM SPSS statistics 25 (New York, United States).

## Results

During the study period, 20,648 patients were referred from 75 general practices to general surgery, gastroenterology or direct to colonoscopy. Of these, 6718 patients had a colonoscopy (Table 1). The probability of having a colonoscopy increased with age (*p* value < 0.001). Female patients were slightly more likely to have a colonoscopy than male patients (33.6% vs 31.2%, *p* value < 0.001), and non-Māori patients were more likely to have a colonoscopy than Māori patients (33.9% vs 23.7%, *p* value < 0.001). Patients with a GP label of HSCan or hospital label of HSCan were more likely to have a colonoscopy than those without an HSCan label.

**Table 1:** Characteristics of patients referred

Characteristics	No colonoscopy		Had colonoscopy		*p* value	Overall
Age group						
30–49	4415	78.0%	1244	22.0%	< 0.001	5659
50–59	2829	67.2%	1381	32.8%		4210
60–69	2829	60.6%	1843	39.4%		4672
70+	3857	63.2%	2250	36.8%		6107
Gender						
Female	7483	66.4%	3790	33.6%	< 0.001	11,273
Male	6447	68.8%	2928	31.2%		9375
Ethnicity						
Non-Māori	11,759	66.1%	6044	33.9%	< 0.001	17,803
Māori	2171	76.3%	674	23.7%		2845
Year						
2015	4936	68.1%	2315	31.9%	0.250	7251
2016	4488	66.8%	2235	33.2%		6723
2017	4506	67.5%	2168	32.5%		6674
High referrer						
Low	4709	67.0%	2321	33.0%	0.290	7030
High	9221	67.7%	4397	32.3%		13,618
HSCan-GP						
Yes	522	47.2%	585	52.8%%	< 0.001	1107
No	13,408	68.6%	6133	31.4%		19,541
HSCan-hospital						
Yes	221	48.8%	232	51.2%	< 0.001	453
No	13,709	67.9%	6486	32.1%		20,195
Overall	13,930	67.5%	6718	32.5%		20,648

As shown in Table 2, after adjustment for the factors shown, the odds ratio of Māori patients having a colonoscopy was 0.66 (95% CI 0.60–0.73). The adjusted odds ratio of the GP practice being a high referrer (i.e., above the median the median referral rate) in having a colonoscopy was 0.94 (95% CI 0.88–1.00). The adjusted odds ratio of a GP label of HSCan and hospital label of HSCan in having a colonoscopy was 2.22 (95% CI 1.92–2.56) and 1.74 (95% CI 1.26–2.42), respectively. After adjustment, gender and year of referral did not have a significant impact on having a colonoscopy.

**Table 2:** Adjusted odds ratio of having a colonoscopy

Factors	*p* value	Odds ratio	95% confidence interval
Age			
(continuous)	< 0.001	1.01	(1.01–1.02)
Gender			
Female	Ref		
Male	< 0.001	0.87	(0.82–0.93)
Year			
(continuous)	0.707	0.99	(0.96–1.03)
Ethnicity			
Non-Māori	Ref		
Māori	< 0.001	0.66	(0.60–0.73)
High referrer			
Low	Ref		
High	0.048	0.94	(0.88–1.00)
HSCan-GP			
No	Ref		
Yes	< 0.001	2.22	(1.92–2.56)
HSCan-hospital			
No	Ref		
Yes	< 0.001	1.74	(1.26–2.42)
Interaction term			
(HSCan-GP × HSCan-Hospital)	0.009	0.57	(0.37–0.87)

Among the patients who had a colonoscopy, 372 (5.5%) were diagnosed with CRC (Table 3). The probability of having CRC increased with age, from 1.5% of patients aged 30–49 years to 9.6% of patients aged 70+ years (*p* value < 0.001). Male patients were more likely to have CRC than female patients (7.1% vs 4.3%). Among patients who had a colonoscopy, 14.7% of patients with a GP label of HSCan were diagnosed with CRC compared to 4.7% of patients who had no GP label of HSCan (*p* value < 0.001), and 17.2% of patients with a hospital label of HSCan were diagnosed with CRC compared to 5.1% of patients who had no hospital label of HSCan (*p* value < 0.001). The proportion of patients who had CRC was similar by ethnicity, year of referral, whether it was direct access colonoscopy, and whether the GP practice was a high referrer.

**Table 3:** Characteristics of patients who had a colonoscopy

Characteristics	No CRC		Had CRC		*p* value	Overall
Age group						
30–49	1225	98.5%	19	1.5%	< 0.001	1244
50–59	1335	96.7%	46	3.3%		1381
60–69	1753	95.1%	90	4.9%		1843
70+	2033	90.4%	217	9.6%		2250
Gender						
Female	3627	95.7%	163	4.3%	< 0.001	3790
Male	2719	92.9%	209	7.1%		2928
Ethnicity						
Non-Māori	5710	94.5%	334	5.5%	0.904	6044
Māori	636	94.4%	38	5.6%		674
Year						
2015	2207	95.3%	108	4.7%	0.056	2315
2016	2095	93.7%	140	6.3%		2235
2017	2044	94.3%	124	5.7%		2168
Direct colonoscopy						
No	2261	93.9%	148	6.1%	0.104	2409
Yes	4085	94.8%	224	5.2%		4309
High referrer						
Low	2202	94.9%	119	5.1%	0.285	2321
High	4144	94.2%	253	5.8%		4397
HSCan-GP						
No	5847	95.3%	286	4.7%	< 0.001	6133
Yes	499	85.3%	86	14.7%		585
HSCan-hospital						
No	6154	94.9%	332	5.1%	< 0.001	6486
Yes	192	82.8%	40	17.2%		232
Overall	6346	94.5%	372	5.5%		6718

After adjustment for age, gender, ethnicity, year of referral, whether it was direct access colonoscopy or not, whether the GP practice was a high referrer or not, hospital label of HSCan and interaction term, the odds ratio of a GP label of HSCan in being diagnosed with CRC was 2.34 (95% CI 1.70–3.22). The adjusted odds ratio of a hospital label of HSCan in being diagnosed with CRC was 2.43 (95% CI 1.18–5.02). The odds ratio of age (for each additional year) and gender (men compared to women) in being diagnosed with CRC was 1.05 (95% CI 1.04–1.06) and 1.67 (95% CI 1.35–2.07), respectively (Table 4). There was no difference in the risk of an underlying CRC for Māori compared to non-Māori or for high referrers compared to low referrers. A subgroup analysis showed that for the 423 patients that were labelled as HSCan by their GP but not by the hospital triage process the underlying risk of CRC was 13.0% (Table 5).

**Table 4:** Adjusted odds ratio of having colorectal cancer

Factors	*P* value	Odds ratio	95% confidence interval
Age			
(continuous)	< 0.001	1.05	(1.04–1.06)
Gender			
Female	Ref		
Male	< 0.001	1.67	(1.35–2.07)
Year			
(continuous)	0.606	1.04	(0.90–1.19)
Ethnicity			
Non-Māori	Ref		
Māori	0.067	1.40	(0.98–2.01)
High referrer			
Low	Ref		
High	0.814	1.03	(0.82–1.29)
Colonoscopy			
FSA and colonoscopy	Ref		
Direct colonoscopy	0.564	0.94	(0.75–1.17)
HSCan-GP			
No	Ref		
Yes	< 0.001	2.34	(1.70–3.22)
HSCan-hospital			
No	Ref		
Yes	0.016	2.43	(1.18–5.02)
Interaction term			
(HSCan-GP × HSCan-Hospital)	0.342	0.65	(0.27–1.57)

**Table 5:** Proportion of patients diagnosed with CRC following colonoscopy by HSCan category

	GP HSCan	No GP HSCan	Totals
Hospital HSCan	31/162 (19.1%)	9/70 (12.9%)	40/232 (17.2%)
No hospital HSCan	55/423 (13.0%)	277/6063 (4.6%)	332/6486 (5.1%)

## Discussion

Colonoscopy is a common diagnostic procedure in patients referred to general surgery or gastroenterology, with 32.5% of patients undergoing the procedure. Thus in the current study, approximately 1.6% (6346/400,000) of patients residing in the Waikato DHB over a 3 year period underwent colonoscopy. This is similar to the 2.0% found in the Netherlands [11], although the proportion who were found to have CRC in the current sample was greater. While this study was conducted before the Covid pandemic reached NZ and before the Waikato DHB introduced free bowel screening for those age 60–74 years, we believe the referral rates for patients with bowel symptoms to our specialist services are unlikely to change substantially. We found older patients and those who had an HSCan label were more likely to receive a colonoscopy. This is unsurprising considering the risk of pathology increases with age. If the clinical picture suggests cancer then these patients should be prioritised. There was a small and probably clinically insignificant difference in the rate of cases accepted for colonoscopy after referral from high referring general practices. This may be due to different risk indicators in patients referred by high referrers. After adjustment for other factors, Māori were 34.0% less likely to have a colonoscopy. While Māori have a lower incidence of CRC than non-Māori, the size of the difference was surprising and needs further investigation. We know that there are differences in the treatment of Māori patients in NZ with CRC [15] e.g., Māori with CRC are less likely to receive surgery or adjuvant chemotherapy.

The study has shown that the conversion rate for CRC following colonoscopy in patients referred from GPs to specialist public hospital care is 5.5%. This is similar to the conversion rate of 4.7% found in the national screening pilot [12] where patients underwent colonoscopy following a positive FIT. This does not mean that 94.5% are negative, as a significant proportion of patients will have adenoma or other relevant pathology—as was found in the screening program [12]. The use of FIT can help rule out CRC in patients presenting in primary care with symptoms [16], however, FIT is not routinely available as a diagnostic tool for NZ GPs. Thus, it is possible that even greater efficiency could be gained in the diagnostic pathway for symptomatic patients which would free up colonoscopy facilities for screening purposes. When considering the underlying likelihood of CRC being found, age was obviously a significant factor, with a steep rise in risk with age from 1.5% in younger patients to 9.6% of patients 70+ years having CRC. Men were much more likely to have CRC with a 7.1% conversion rate compared with women at 4.3%. These findings support the guidance for referral. However, we know that there is also an increase in the incidence of CRC in younger patients in NZ [17] and if cases are not to be missed it may still be worthwhile offering colonoscopy to younger patients in order to exclude cancer. While there was no difference in the likelihood of Māori undergoing colonoscopy having CRC (5.6% vs 5.5% in non-Māori) we know the incidence of CRC in Māori is less than in non-Māori. If Māori rates of colonoscopy were similar to non-Māori we may find that the positivity rate would fall in line with the known lower incidence of CRC in Māori. The characteristics of the general practice where patients were registered did not seem to influence the conversion rate—thus those patients referred for direct colonoscopy did not differ, and there was no difference in the rate of CRC for high referrers compared to low referrers. However, if the GP had indicated an HSCan and a colonoscopy was carried out, then the conversion rate was 14.7%. While the rate in those deemed an HSCan by the hospital specialist team was higher at 17.2%, this was based on only 232 cases. Seventy two percent (423/585) of GP HSCan patients were downgraded resulting in 55/423 (13.0%) patients deemed as having an HSCan by their GP having an unnecessary delay in diagnosis due to the hospital triage process. We could argue that the sensitivity and specificity of a GP identification of an HSCan is such that all these patients should be offered an urgent colonoscopy. The poor outcomes in NZ from CRC have been linked to late diagnosis and any opportunity to expedite a diagnosis rapidly could be considered worthwhile.

## Strengths and limitations

A study strength is that we have outcome data on over 6000 colonoscopy cases following referral from GPs. We did not have data on patients who were invited to have a colonoscopy but did not attend. Study data included both patient and GP characteristics. A weakness is that we did not have complete data on symptoms, or the reason for referral. In addition, outcome data only included a diagnosis of CRC derived from the Cancer Registry. Therefore, we did not have information on other pathology found at colonoscopy.

## Implications

The implications of these findings for policy include the need for NZ Bowel Cancer Guidelines to reassess the use of the HSCan and 2 week wait rule for patients deemed at high suspicion of cancer by their GP. We would argue that all patients deemed at high risk by their GP should be offered timely colonoscopy and that further delay by an additional triage step by the hospital in the referral pathway is unnecessary. We also believe that it is timely for NZ to review their guidelines for diagnosis in the light of the UK NICE guidance [18] and introduce the option of a FIT test in general practice to help rule out the need for referral for colonoscopy. Finally, given the poor outcomes for Māori following a diagnosis of CRC, the finding of a lower use of colonoscopy is of concern.

## Conclusions

Almost six percent of colonoscopies in symptomatic patients referred by general practitioners result in a finding of CRC. The likelihood of cancer increases with age and is greater in men. If the GP identifies a high risk of cancer then the likelihood of a positive colonoscopy is almost 15%, suggesting that these patients should be routinely prioritised without the need for further hospital triage. Further research is needed to understand why Māori are less likely to receive a colonoscopy following referral from general practice.

## Data Availability

The data analysed for the current study are not publicly available for ethical reasons. All data relevant to the study are included in the article. Anonymised data can be made available from the corresponding author on request.

## Abbreviations

NZ: New Zealand
CRC: Colorectal cancer
GP: General practitioner
DHB: District Health Board
MOH: Ministry of Health
HSCAn: High suspicion of cancer
UK: United Kingdom
FSA: First Specialist Assessment
NHI: National Health Index
OR: Odds ratio
CI: Confidence interval
FIT: Faecal Immunochemical test
